# Gesundheitliche Folgen sozialer Isolation: Qualitative Studie zu psychosozialen Belastungen und Ressourcen älterer Menschen im Zusammenhang mit der COVID-19-Pandemie

**DOI:** 10.1007/s00103-021-03281-5

**Published:** 2021-02-02

**Authors:** Franziska D. Welzel, Katja Schladitz, Franziska Förster, Margrit Löbner, Steffi G. Riedel-Heller

**Affiliations:** Institut für Sozialmedizin, Arbeitsmedizin und Public Health (ISAP), Medizinische Fakultät, Universität Leipzig, Philipp-Rosenthal-Straße 55, 04103 Leipzig, Deutschland

**Keywords:** SARS-CoV-2, Psychosoziale Gesundheit, Hohes Lebensalter, Soziale Isolation, Bewältigung, SARS-CoV-2, Psychosocial health, Late life, Social isolation, Coping

## Abstract

**Hintergrund:**

Mit dem SARS-CoV-2-Ausbruchsgeschehen („Severe acute respiratory syndrome coronavirus type 2“, COVID-19) ist es zu einer Verunsicherung über Erkrankungsrisiko und Folgen der Virusinfektion in der Bevölkerung gekommen. Ältere Menschen gelten als Risikogruppe für schwere Infektionsverläufe und wurden im besonderen Maße zu sozialer Distanzierung aufgerufen. Gleichzeitig wurde die Sorge geäußert, dass sich Erkrankungsrisiko und soziale Isolation negativ auf die psychische Gesundheit älterer Menschen auswirken würden.

**Ziele der Arbeit:**

Erfassung von psychosozialen Belastungen, vorhandenen Bewältigungsstrategien, Unterstützungsbedarfen und Kohärenzerleben älterer Menschen im Zusammenhang mit dem COVID-19-Ausbruchsgeschehen.

**Material und Methoden:**

Die Studie folgt einem qualitativen Untersuchungsdesign. Zwischen Mai und Juni 2020 wurden telefonische Interviews mit 11 älteren Personen (70+) durchgeführt. Die Durchführung der Interviews erfolgte leitfadengestützt. Die Daten wurden mittels Audioaufzeichnung festgehalten, transkribiert und inhaltsanalytisch nach Mayring und Fenzl (2019) unter Nutzung von MAXQDA ausgewertet.

**Ergebnisse:**

Die Probanden waren im Durchschnitt 74,8 Jahre alt. Bei den Befragten zeigte sich ein überwiegend stabiles Befinden und gutes Zurechtkommen mit dem COVID-19-Geschehen. Als wesentliche Ressourcen wurden Lebenserfahrung, frühere bewältigte Krisen, eine optimistische Grundhaltung und Einsicht in die Notwendigkeit der Maßnahmen genannt. Externe Unterstützungsangebote seien kaum in Anspruch genommen worden. Das Schließen seniorenspezifischer Treffpunkte wurde kritisch bewertet.

**Diskussion:**

Ältere Menschen scheinen sich ihre psychosoziale Gesundheit trotz COVID-19-Pandemie überwiegend zu erhalten. Die Bedeutsamkeit mentaler Ressourcen älterer Menschen für die Unterstützung jüngerer Generationen bleibt bisher unerkannt.

**Zusatzmaterial online:**

Zusätzliche Informationen sind in der Online-Version dieses Artikels (10.1007/s00103-021-03281-5) enthalten.

## Abstract (en)

**Background:**

The SARS-CoV-2 pandemic led to a general uncertainty about risk and consequences of the disease. Older adults are specifically vulnerable with regard to severe courses of the disease and have been particularly encouraged to self-isolate during the pandemic. Subsequently, expressions of concern have been raised regarding the negative impact of disease risk and quarantine on the mental health of older people.

**Objectives:**

Assessment of psychosocial stress, coping strategies, need for support, and sense of coherence of older people during the COVID-19 pandemic.

**Materials and methods:**

The study follows a qualitative research design. Between May and June 2020 *N* = 11 guided telephone interviews were conducted with older adults (70+ years). Telephone interviews were recorded by audio tape and fully transcribed. A qualitative content analysis was performed according to Mayring and Fenzl (2019) using MAXQDA.

**Results:**

Participants were 74.8 years old on average. Participants showed predominantly good psychosocial health and functional coping strategies. Life experience, an optimistic attitude, understanding of the necessity of restrictions, and previous crises that have been mastered were the most important resources during the COVID-19 pandemic for older adults. Offers of support were rarely used. Participants were critical of the closing of centers or meeting points for older people.

**Conclusion:**

Older adults appear to be able to preserve their mental wellbeing during the COVID-19 pandemic. The relevance of mental resources of older adults for supporting younger generations seems to be unrecognized.

## Hintergrund

Quarantäne- und Isolationsmaßnahmen zur Eindämmung von Infektionsgeschehen gehen mit negativen Auswirkungen auf die psychosoziale Gesundheit einher [1, 2]. Aktuelle internationale Studien verweisen auf ähnliche Konsequenzen einschränkender Maßnahmen im Zusammenhang mit dem SARS-CoV-2-Ausbruchsgeschehen (COVID-19; [3, 4]). Ältere Menschen und Personen mit Vorerkrankungen gelten als spezifisch vulnerabel für schwere Infektionsverläufe im Falle einer Ansteckung mit dem SARS-CoV-2-Virus und wurden im besonderen Maße zu sozialer Distanzierung im Zuge der COVID-19-Pandemie aufgerufen [5–7]. In der Folge wurden negative Auswirkungen von sozialer Distanzierung und Isolation auf die psychische Gesundheit älterer Menschen befürchtet, zusammen mit der Sorge vor einer erheblichen Versorgungslücke in dieser Altersgruppe [8, 9].

Mittlerweile liegen erste Ergebnisse zur psychischen Gesundheit älterer Menschen im Zusammenhang mit der COVID-19-Pandemie vor. In einer repräsentativen Studie der Universität Leipzig wurden Personen im Alter zwischen 65 bis 94 Jahren zu ihren Einstellungen zur COVID-19-Pandemie, zu Gesundheitsschutzmaßnahmen und ihrer psychosozialen Gesundheit befragt [10]. Ein Großteil der befragten Senioren äußerte Besorgnis über die COVID-19-Pandemie und zeigte Verständnis für die einschränkenden Maßnahmen. Gleichzeitig zeigte sich die psychosoziale Gesundheit älterer Menschen durch die Pandemie und den COVID-19-Lockdown erstaunlich unverändert. Die Autoren schlussfolgern, dass sich ältere Menschen während des COVID-19-Lockdowns überwiegend als resilient und psychisch stabil erwiesen.

Psychische Belastungen waren dagegen in spezifischen Subgruppen älterer Menschen erhöht (z. B. bei fehlender sozialer Unterstützung; [10]). Im COVID-19 Snapshot Monitoring (COSMO), einer wiederholten querschnittlichen Erfassung einer repräsentativen Stichprobe der deutschen Bevölkerung während des COVID-19-Geschehens, zeigten sich tendenziell höhere Werte in der allgemeinen Lebenszufriedenheit sowie ein geringeres situatives Belastungserleben unter den 65- bis 74-Jährigen im Vergleich zu jüngeren Altersgruppen [11]. Weitere Hinweise darauf, dass ältere Menschen in ihrem psychischen Wohlbefinden während des COVID-19-Geschehens vermutlich nicht stärker belastet sind oder weniger beeinträchtigt sind als jüngere Altersgruppen, liefern internationale Studien [3, 12].

Die Ergebnisse können aus der Perspektive psychologischer Modelle von Gesundheit und Krankheit betrachtet werden. Nach dem transaktionalen Stressmodell von Lazarus und Folkman [13] ergibt sich psychologischer Stress aus einem interaktiven Prozess zwischen Merkmalen der Umwelt und des Individuums. Das Erleben von psychologischem Stress in einer Situation wie der des COVID-19-Ausbruchsgeschehens ist demnach maßgeblich von der kognitiven Bewertung der Situation (als irrelevant, positiv oder gefährlich) und der Einschätzung verfügbarer Bewältigungsmöglichkeiten des Individuums abhängig [13]. Im Zusammenhang mit dem personalen Bewältigungserleben kann ferner das Konzept des Kohärenzgefühls nach Antonovsky [14] als wesentliche Ressource verstanden werden. Das Kohärenzgefühl umfasst das Vertrauen einer Person darin, dass (a) Ereignisse des Lebens erklärbar sind (Verstehbarkeit), (b) dass man Ressourcen zur Bewältigung anfordernder Situationen zur Verfügung hat (Handhabbarkeit), (c) dass sich Anstrengungen lohnen und dass das eigene Leben bedeutsam ist (Bedeutsamkeit; [14]).

Bisher ist jedoch wenig dazu bekannt, welche Themen ältere Menschen zur Erhaltung ihrer Gesundheit in der COVID-19-Pandemie selbst als relevant erachten. Es fehlt an Informationen zu den konkreten Bewertungen, Bewältigungsstrategien und Versorgungsbedarfen, die aus Sicht älterer Menschen für den Umgang mit der aktuellen Situation wichtig sind. Für die Erfassung subjektiver Konstrukte von Gesundheit eignen sich insbesondere qualitative Untersuchungsmethoden. Diese ermöglichen die Erforschung von neuen Forschungsgegenständen mit einem hohen Maß an Tiefe und Flexibilität sowie die Exploration neuer Aspekte, die sich im Interviewverlauf ergeben [15].

Ziel der vorliegenden qualitativen Arbeit war es, die psychosoziale Belastung, vorhandene Bewältigungsstrategien, relevante Versorgungsbedarfe und das Kohärenzerleben älterer Menschen im Zusammenhang mit dem COVID-19-Ausbruchsgeschehen zu erfassen.

## Material und Methoden

Die vorliegende Studie folgt einem qualitativen Untersuchungsdesign in Form von telefonisch erhobenen Einzelinterviews. Die Durchführung der Interviews erfolgte leitfadengestützt. Der teilstandardisierte Interviewleitfaden wurde im Rahmen einer qualitativen Forschungswerkstatt entwickelt, in welcher auf Basis des transaktionalen Stressmodells [13] sowie des Salutogenesemodells [14, 16] deduktiv Leitfragen abgeleitet und Konsens hergestellt wurde. Die Teilnehmenden wurden über Stressoren, ihre Bewertung dieser Stressoren sowie erlebte Einschränkungen und Belastungen im Zusammenhang mit der COVID-19-Pandemie befragt. Weitere Interviewinhalte umfassten die Einschätzung vorhandener Ressourcen, eingesetzter Bewältigungsstrategien zur Aufrechterhaltung des Wohlbefindens und die Bewertung von Sinnhaftigkeit und Chancen im Zuge der COVID-19-Pandemie. Abschließend wurden mögliche Versorgungs- und Unterstützungsbedarfe für ältere Menschen in der COVID-19-Pandemie erfragt.

### Interviewteilnehmer/innen

Zwischen Mai und Juni 2020 wurden 11 telefonische Interviews mit 6 Frauen und 5 Männern durchgeführt. Die Rekrutierung erfolgte aus dem Teilnehmerkreis zweier Gruppendiskussionen im Oktober 2019 im Rahmen des geschlechtsspezifischen Alterns (AgeDifferent-Studie), welche ihr Einverständnis zu weiteren qualitativen Studienbefragungen gegeben hatten. Die Probanden wurden initial über den Seniorenbeirat der Stadt Leipzig, Aushänge in Supermärkten, Sportvereine für Senioren sowie Senioreneinrichtungen angesprochen. Soziodemografische Daten wurden mittels eines kurzen Fragebogens schriftlich erfasst.

### Einhaltung ethischer Richtlinien

Die vorliegende Studie wurde in Übereinstimmung mit der Deklaration von Helsinki sowie unter Beachtung der Leitlinie für die gute klinische Praxis durchgeführt. Vor der telefonischen Befragung wurden die Probanden schriftlich und mündlich über die Inhalte informiert und willigten schriftlich in die Studienteilnahme ein. Für das Projekt liegt ein Ethikvotum durch die Ethikkommission der Medizinischen Fakultät der Universität Leipzig vor (194/20-ek) [17].

### Durchführung und Auswertung

Die Interviews wurden von 2 geschulten wissenschaftlichen Mitarbeiterinnen durchgeführt und hatten eine Dauer von 29–85 min. Die Interviews wurden mittels Audioaufzeichnung festgehalten, vollständig transkribiert und inhaltsanalytisch nach Mayring und Fenzl [18] unter Nutzung der Software MAXQDA 2018 (VERBI GmbH, Berlin, Deutschland) ausgewertet. 2 Mitarbeiterinnen aus dem Fachbereich Soziologie und Psychologie codierten die Transkripte und folgten in der Ableitung des Codierschemas einer kombinierten deduktiven und induktiven Vorgehensweise (deduktiv aus dem modellbasierten Interviewleitfaden sowie induktiv aus dem Interviewmaterial). Konsens wurde mittels gegenseitiger Abstimmung bei den Zwischenschritten (Probecodierung, Revision, Recodierung sowie Codierung) sowie einer finalen Gruppendiskussion im Forschungsteam hergestellt. Die Auswertung erfolgte anonymisiert.

## Ergebnisse

Die Teilnehmenden waren im Durchschnitt 74,8 Jahre alt (*SD* = 2,7). Soziodemografische Charakteristika sind in Tab. 1 abgebildet. Eine Übersicht der abgeleiteten Themenkategorien sowie Schlüsselzitate sind in Tab. Z1 (siehe Onlinematerial) aufgeführt. Die Ergebnisse werden nachfolgend entsprechend der Hauptkategorien (HK) gegliedert dargestellt.

**Table 1:** 

	Interviewteilnehmer/innen	
*N*	11	
*Geschlecht, n*		
Frauen	6	
Männer	5	
*Alter, Mittelwert (SD)*	74,8	(2,7)
*Bildung, n (%)*		
Niedrig	0	(0)
Mittel	4	(36,4)
Hoch	7	(63,6)
*Wohnsituation, n (%)*		
Allein	6	(54,5)
Mit Ehepartner	5	(45,5)
*Familienstand, n (%)*		
Verheiratet	5	(45,5)
Geschieden	3	(27,3)
Verwitwet	3	(27,3)

### HK1 Infektions- und Isolationserfahrungen

Keine/r der Befragten berichtete von einer eigenen Infektionserfahrung mit COVID-19. Eine Teilnehmerin sei in offiziell angeordneter Quarantäne gewesen, ein anderer Teilnehmer habe sich bei einem Schnupfen in selbst gewählte „vorbeugende“ Quarantäne begeben. Alle Übrigen verneinten eigene offiziell angeordnete Quarantäne- oder Isolationserfahrungen. 2 Personen gaben an, jemanden aus dem Umfeld zu kennen, der eine Quarantäne bzw. Isolation einhalten musste.

### HK2 Bewertung des COVID-19-Geschehens

#### Zustandsbewertung

Zum Zeitpunkt der Interviews (Mai/Juni 2020) berichteten bis auf eine Teilnehmerin alle Befragten ein insgesamt gutes Befinden und gutes Zurechtkommen in der aktuellen Situation. Eine Teilnehmerin gab eine depressive Grundstimmung an. Alle Teilnehmenden erzählten rückblickend auf die Anfangszeit des Lockdowns im März und April, dass sie die Entwicklung intensiv verfolgt und diskutiert hätten und es in vielen Fällen eine Anpassungsphase mit teils starken Verunsicherungen und in einem Fall mit einem intensiven Stimmungstief gegeben habe.

#### Ängste/Sorgen

Die Interviewten berichteten vor allem von Ängsten und Sorgen in Bezug auf nahestehende Menschen (Kinder, Enkelkinder, Freundeskreis) und hinsichtlich der langfristigen gesellschaftspolitischen und wirtschaftlichen Folgen. Der größte Teil der Befragten berichtete, sich altersbedingt als Risikogruppe zu sehen, aber sich wenig Sorgen um eine eigene Gefährdung zu machen: zum einen, weil sie sich durch das Einhalten der Hygieneregeln gut geschützt sähen, zum anderen, weil sie eine akzeptierende Haltung der Möglichkeit einer Ansteckung gegenüber entwickelt hätten.

#### Bewertung einschränkender Maßnahmen

Die Maßnahmen empfänden alle Befragten als Einschränkung und Belastung vor allem hinsichtlich Freizeit- und Sportaktivitäten, Reisen und sozialer Kontakte. Man habe sich aber mit der Zeit arrangieren können und passe seine Gewohnheiten an. Der Alltag sei insgesamt unbequemer geworden, man könne aber mit den Einschränkungen leben. Der Wegfall von Tagesstrukturen, z. B. aufgrund der Schließung von Seniorentreffs oder Volkshochschulen, wurde teilweise als belastend, teilweise auch als entlastend erlebt (man müsse nicht mehr ständig etwas unternehmen). Soziale Kontakte hätten i. d. R. abgenommen, da die Anlässe dafür fehlten (z. B. Sporttreff) bzw. durch die Eindämmungsmaßnahmen untersagt waren. Sozial isoliert würden sich vor allem diejenigen fühlen, die vorher schon wenig familiäre oder freundschaftliche Kontakte hatten. Die medizinische Versorgung sei von den Befragten nur geringfügig als eingeschränkt erlebt worden. Finanzielle Einschränkungen habe niemand erfahren.

### HK3 Bewertung vorhandener Ressourcen

#### Eigene Ressourcen

Die Befragten benannten als interne Ressourcen, die ihnen zur Verfügung stünden, in erster Linie ihre Lebenserfahrung und bewältigte Krisen (u. a. Nachkriegszeiterfahrungen mit Mangelerscheinungen, Sozialisationserfahrungen in der DDR mit planwirtschaftlich bedingten Engpässen, die Überwindung beruflicher Schwierigkeiten). Auch mentale Ressourcen wie eine optimistische Grundhaltung, Einsicht in die Notwendigkeit der Maßnahmen sowie Ruhe und Entspanntheit wurden als sehr wichtig benannt. Die Befragten hoben rückblickend für die Zeit der strengen Ausgangsbeschränkungen die Relevanz guter Wohnbedingungen hervor, die ihnen geholfen hätten: z. B. Kleingarten, Park, Balkon oder Terrasse.

#### Externe Ressourcen

Die Interviewten gaben an, dass sie i. d. R. keine externen Ressourcen zur Unterstützung in Anspruch nehmen mussten, sondern ihre Alltagsbesorgungen selbst oder mit dem Partner erledigen konnten. Nach ihrer Aussage seien grundsätzlich Familienangehörige (z. B. Kinder und Enkelkinder) die wichtigste Quelle potenzieller Unterstützung, aber auch das Angebot der Nachbarschaftshilfe durch Aushänge im Haus oder persönliches Ansprechen wurde als erfreuliche Geste wahrgenommen. Familiäre und freundschaftliche Kontakte seien als Quelle der emotionalen Unterstützung wichtig. Dienstleistungen wie Lieferdienste oder Unterstützung durch Organisationen seien hingegen so gut wie nie in Anspruch genommen worden: Apothekenlieferdienste und Onlineeinkäufe sowie in einem Fall eine über das Gesundheitsamt organisierte Einkaufshilfe seien nur teilweise genutzt worden. Dennoch wurde es als wichtig benannt, dass es diese Optionen gäbe.

### HK4 Eingesetzte Bewältigungsstrategien

#### Aufrechterhaltung des Wohlbefindens

Die Interviewteilnehmenden gaben an, verschiedene Strategien zur Aufrechterhaltung ihres seelischen und körperlichen Wohlbefindens zu nutzen. Sie hoben hervor, dass es wichtig sei, die für sich persönlich geeigneten Strategien zu finden und aktiv einzusetzen. Meist wurden Strategien genannt, die schon vorher genutzt und nun auf die neue Situation angepasst wurden: So wurden z. B. als Ersatz für Sportgruppen Gymnastik zu Hause, Laufen, Radfahren oder Spazieren im Park sowie Gartenarbeit aufgezählt. Auch für das seelische Wohlbefinden seien etablierte Strategien adaptiert und bewusst eingesetzt worden: Besonders häufig wurde der Aufenthalt in der Natur genannt, daneben Lesen, Musikhören als Konzertersatz sowie allgemein Ablenkung und Aufrechterhaltung sozialer Kontakte.

#### Rolle der Medien

Die meisten Befragten würden im Rahmen ihrer Bewältigungsstrategien vor allem klassische Medien (z. B. Tageszeitungen, Fernsehen, Radio) und seltener neue Medien (Internet) zur Informationsgewinnung und zur Unterhaltung nutzen. Das Internet würde eher zur Aufrechterhaltung sozialer Kontakte sowie für alltagspraktische Zwecke genutzt (z. B. Onlinebestellungen und -banking). Einige Befragte erzählten, dass sie ersatzweise angebotene Onlinealternativen für Sportkurse nutzen würden. Insgesamt ergab sich bei der Nutzung neuer Medien ein breitgefächertes Bild: Einige Teilnehmende gaben an, kein Internet zu nutzen, andere berichteten von intensiver Internetnutzung. Auch für die Rolle neuer Medien in der medizinischen Versorgung ergab sich ein vielfältiges Bild: Einige Befragte äußerten große Skepsis (z. B. wegen der hohen Bedeutung persönlicher Nähe), andere sahen hierin eine Chance, medizinische Versorgungsstrukturen zu ergänzen und medizinisches Personal zu entlasten.

### HK5 Kohärenzsinn

#### Verstehbarkeit

Die Befragten gaben an, dass sie die Maßnahmen im Wesentlichen für sinnvoll hielten und die Gründe dafür verstünden. Sie äußerten grundsätzlich Vertrauen in die Wissenschaft und hätten Verständnis dafür, dass es einen Lernprozess hinsichtlich COVID-19 gäbe, in dessen Verlauf auch Einschätzungen überarbeitet werden müssten und Entscheidungen getroffen würden, die im Nachhinein als fehlerhaft betrachtet würden. Schwierig wäre vor allem, die Übersicht zu behalten, was zu welchem Zeitpunkt und in welchem Bundesland gelten würde. Die Uneinheitlichkeit zwischen den Bundesländern und die schnellen Lockerungen wurden als Problem benannt. Auch empfanden einige Befragte starkes Unverständnis gegenüber den aufkommenden Verschwörungstheorien bezüglich COVID-19 und dem teilweise lockeren Umgang jüngerer Menschen mit den Maßnahmen, welchen sie als verantwortungslos empfänden.

#### Handhabbarkeit

Das Wichtigste sei aus Sicht der Befragten, die offiziellen Regeln des Robert Koch-Instituts und der Bundes- und Landesregierungen einzuhalten, um sich und andere zu schützen und die Verbreitung einzudämmen, und diese Einhaltung auch im Umfeld zu propagieren.

#### Chancen der Krise/Bedeutsamkeit

Die Befragten äußerten ein differenziertes Bild hinsichtlich potenzieller Chancen der COVID-19-Pandemie: Für sich selbst oder das nähere familiäre oder bekanntschaftliche Umfeld würden kaum positive Aspekte gesehen (eine Befragte fände es gut, wenn die Nachbarschaftshilfe die Pandemie überdauere). Mit Blick auf gesellschaftliche Chancen wurde in Erwägung gezogen, ob wirtschaftliche und politische Fehlentwicklungen hinterfragt werden würden (z. B. Aspekte der Globalisierung oder das Prinzip „höher, schneller, weiter“ in der Wirtschaft) und strategische Verbesserungen in Gang kämen (z. B. nachhaltige Produktion, Entschleunigung, Wertschätzung für Lehrer/innen und Erzieher/innen). Ein Großteil der Befragten ging davon aus, dass sich gesamtgesellschaftlich keine langfristigen positiven Veränderungen ergeben würden. Die meisten Befragten hätten noch nicht darüber nachgedacht, inwiefern sie einen eigenen Beitrag im Zuge des COVID-19-Geschehens leisten könnten. Teilweise wurde emotionale Unterstützung von Familienangehörigen und Bekannten als eigener Beitrag genannt.

### HK6 Versorgungs- und Unterstützungsbedarfe

#### Informationsbedarfe

Alle Studienteilnehmenden fühlten sich grundsätzlich gut informiert und würden für sich keine Bereiche sehen, wo sie Unterstützungsbedarf feststellten. Eine Ausnahme sei eine tagesaktuelle Übersicht, welche Regelungen in welchem Bundesland jeweils aktuell gelten, da es schwer sei, den Überblick bei den Lockerungen und Beschränkungen zu behalten. Der Informationsbedarf könne jedoch bei sehr zurückgezogenen oder kognitiv eingeschränkteren Menschen in ihrer Altersgruppe anders sein – diese seien zu Informationszwecken z. B. über Unterstützungsangebote vor allem über Fernsehen, Radio und Zeitungen bzw. Zeitschriften erreichbar, auch Aushänge in Supermärkten oder Hausfluren wurden befürwortet. Internet, Postwurfsendungen oder Telefonie würden für diese Zielgruppe i. d. R. als wenig geeignet empfunden.

#### Unterstützungsbedarfe

Hinsichtlich praktischer Unterstützung wurde das Problem der Einsamkeit benannt. Allerdings sei dies etwas, was grundsätzlich bestünde und durch die Pandemie in die öffentliche Aufmerksamkeit geraten sei. Hier könne es mehr niedrigschwellige Angebote geben. Aus Sicht der Befragten sei es sehr negativ gewesen, die bestehenden Seniorentreffpunkte zum Infektionsschutz zu schließen und Besuche in Pflegeheimen zeitweise zu untersagen. Die praktischen Angebote der Nachbarschaftshilfe würden ihrer Meinung nach ausreichen. Es gäbe allerdings Menschen, die sich so sehr zurückzögen oder sich scheuten, Hilfe anzunehmen, dass man sie kaum erreichen könne.

## Diskussion

Ziel der vorliegenden Arbeit war es, die psychosoziale Belastung, Bewältigungsstrategien, Informations- und Unterstützungsbedarfe und das Kohärenzerleben älterer Menschen im Zusammenhang mit dem COVID-19-Ausbruchsgeschehen mithilfe qualitativer Methoden zu erfassen.

Im Ergebnis zeigte sich ein überwiegend stabiles Befinden und gutes Zurechtkommen mit dem COVID-19-Geschehen bei den meisten Älteren der Studie. Als wesentliche Ressourcen wurden Lebenserfahrung, frühere bewältigte Krisen, eine optimistische Grundhaltung und Einsicht in die Notwendigkeit der Maßnahmen genannt. Externe Unterstützungsangebote seien kaum in Anspruch genommen worden. Das Schließen seniorenspezifischer Treffpunkte wurde insbesondere vor dem Hintergrund einer zunehmenden Einsamkeit älterer Menschen kritisch bewertet.

### Psychosoziale Gesundheit

Ältere Menschen, die vor COVID-19 über eine gute psychosoziale Gesundheit verfügten, erhalten sich diese auch während der Pandemie und den damit einhergehenden einschränkenden Maßnahmen. Dieses Ergebnis deckt sich mit aktuellen Studien, die die psychosoziale Gesundheit älterer Menschen im Zuge der COVID-19-Pandemie untersuchten [3, 10, 12]. Aus der Literatur lässt sich für das COVID-19-Ausbruchsgeschehen bisher ableiten, dass negative Auswirkungen von Quarantäne- und Isolationserfahrungen auf die psychische Gesundheit in stärkerem Maße jüngere Generationen [3] sowie spezifische Subgruppen (v. a. Personen mit fehlender sozialer Unterstützung oder hoher Krankheitslast) [10] betreffen. Die gefundenen Ergebnisse können auf Basis des transaktionalen Stressmodells [13] unter Berücksichtigung individuell vorliegender Belastungen und vorhandener Bewältigungsressourcen interpretiert werden. Während ältere Menschen stärker als Risikogruppe für schwere Infektionsverläufe gelten, erleben jüngere Menschen (<50 Jahre) im Zuge des COVID-19-Geschehens in stärkerem Ausmaß ein situatives Belastungserleben (z. B. durch akute Mehrfachbelastung im Zusammenhang mit Kinderbetreuung, Unterstützung älterer Familienangehöriger oder finanzielle Einbußen durch Kurzzeitjobs) [11]. Jüngere Menschen haben zudem ein etwas höheres Bewegungs- und Aktivitätsbedürfnis als ältere Menschen [19, 20] und erleben im Zusammenhang mit einschränkenden Maßnahmen womöglich auch stärkere negative Auswirkungen auf ihre psychosoziale Gesundheit.

### Ressourcen und Kohärenzerleben

Nach dem Modell von Lazarus und Folkman [13] ist das Belastungserleben zudem abhängig von der Bewertung der eigenen Bewältigungsressourcen. Unsere Ergebnisse zeigen, dass ältere Menschen insbesondere auf ihre Lebenserfahrung und bewältigte schwierige Lebensereignisse zurückgreifen, um sich ihre optimistische Einstellung und ihr Befinden auf stabilem Niveau zu erhalten. Ereignisse als verstehbar und handhabbar zu erleben und das eigene Handeln sowie das eigene Leben als bedeutsam zu begreifen, sind wichtige Ressourcen für den Umgang mit herausfordernden Situationen [14]. Diese Aspekte (Verstehbarkeit, Handhabbarkeit und Bedeutsamkeit) werden unter dem Konzept des Kohärenzerlebens zusammengefasst [14, 16]. Die Maßnahmen zur Viruseindämmung wurden in dieser Untersuchung von den befragten Personen überwiegend als verstehbar und handhabbar erlebt. Die Bedeutsamkeit eigenen Handelns (z. B. auch in Form der emotionalen Unterstützung anderer) und die Wichtigkeit des eigenen Lebens für andere wurden von der Zielgruppe nur wenig reflektiert. Ein aktuelles Review zum Konzept der Wichtigkeit beschreibt das Erleben einer Person, für jemand anderen von Bedeutung zu sein, als einen wichtigen protektiven Faktor und als wesentlich für funktionales Coping und Resilienz im Zusammenhang mit der COVID-19-Pandemie [21].

Vor dem Hintergrund, dass sich ältere Menschen in der COVID-19-Pandemie vorwiegend psychisch stabil zeigen, sollten die vorhandenen emotionalen bzw. mentalen Ressourcen dieser Altersgruppe eine größere gesellschaftliche Aufmerksamkeit erfahren. Die Erfahrungen aus wiederholten bewältigten Krisen im Laufe eines Lebens können insofern im Sinne einer gesellschaftlichen Ressource verstanden werden. Eine stärkere Einbindung dieser Ressource hinsichtlich der Gestaltung und Durchführung psychosozialer Unterstützungsangebote könnte einerseits belasteten Subgruppen und jüngeren Generationen zugutekommen sowie gleichzeitig das Erleben der Bedeutsamkeit älterer Menschen positiv beeinflussen.

### Informationsbedarfe

Für Informations- und Unterhaltungszwecke wurden in unserer Stichprobe mit älteren Menschen v. a. traditionelle Medien (Tageszeitungen, Fernseher, Radio) genutzt. Große individuelle Unterschiede fanden sich hinsichtlich des Einsatzes und der Bewertung des Mediums Internet für Informationsgewinnung, Unterhaltung sowie Kontaktpflege und medizinische Versorgung. Gegenwärtige Studien zum Mediengebrauch zeigen jedoch einen stetigen Zuwachs der Internetnutzung in der Zielgruppe älterer Menschen in den letzten Jahren [22, 23]. Eine Ansprache älterer Menschen für Gesundheitsthemen sollte daher vor dem Hintergrund großer interindividueller Unterschiede stets über mehrere Medien erfolgen.

### Unterstützungsbedarfe

Unterstützungsbedarfe wurden von unserer Zielgruppe vorrangig hinsichtlich des situationsüberdauernden Aspektes der Einsamkeit älterer Menschen gesehen. Aus der Literatur ist bekannt, dass die Determinanten von Einsamkeit (u. a. Verlusterfahrungen) insbesondere im höheren Lebensalter zunehmen [24]. Dabei scheint weniger die objektive soziale Isolation als vielmehr die subjektive Wahrnehmung und Bewertung, keine oder nur wenige bedeutsame Kontakte zu haben, mit negativen Effekten auf die psychosoziale Gesundheit assoziiert zu sein [25]. Aus den vorliegenden Ergebnissen lässt sich ableiten, dass zur Erhaltung der psychosozialen Gesundheit älterer Menschen im Rahmen der COVID-19-Pandemie der Aufrechterhaltung persönlicher Kontakt- und Begegnungsmöglichkeiten unter Beachtung geeigneter Hygienemaßnahmen eine wesentliche Bedeutung zuzukommen scheint.

## Stärken und Limitationen

Die vorliegende Studie folgt einem qualitativen Studiendesign. Qualitative Methoden sind ergebnisoffener als quantitative Methoden und ermöglichen die Entdeckung neuer oder bisher unbeachteter Aspekte. Eine Stärke der Studie liegt daher insbesondere darin, die für ältere Personen relevanten Gesundheitsthemen im Zusammenhang mit COVID-19 aus der Perspektive der Zielgruppe heraus zu erfassen.

Eine Einschränkung bei qualitativen Studien ist die meist geringe Stichprobengröße, wodurch die Generalisierbarkeit der Ergebnisse begrenzt ist. Als Kriterium für die Verallgemeinerung wird bei qualitativen Studien jedoch weniger die Anzahl von Interviews per se, sondern die theoretische Sättigung bzw. Saturiertheit herangezogen [26]. In der Literatur wird üblicherweise davon ausgegangen, dass Sättigung vorhanden ist, wenn einerseits keine neuen Aspekte mehr benannt werden und andererseits auch seltene bzw. Extrempositionen benannt wurden [26, 27]. In der vorliegenden Studie wurde das Forschungsfeld aus unterschiedlichen Perspektiven heraus (Männer und Frauen, allein Lebende und in Gemeinschaft Lebende) untersucht. Dabei wurden ähnliche Inhalte von den Probanden benannt, aber auch minoritäre Positionen erfasst. Entsprechend gehen wir davon aus, dass wir die wesentlichen Inhalte erfassen konnten und eine ausreichende Sättigung erreicht haben.

Dennoch ist limitierend anzumerken, dass ausschließlich Personen aus dem Stadtgebiet Leipzig befragt wurden und eine Verallgemeinerung auf andere Regionen womöglich begrenzt ist. Ferner wiesen alle befragten Probanden einen mittleren oder hohen Bildungsstand auf und zeichneten sich durch einen überwiegend guten bis mäßigen Gesundheitszustand aus. Die Ergebnisse müssen insofern vor dem Hintergrund einer eingeschränkten Generalisierbarkeit insbesondere für die Subgruppen älterer Menschen mit hoher Krankheitslast, niedrigem Bildungsniveau und aus ländlichen Gebieten betrachtet werden. Andererseits weist die Stichprobe eine gute Balance hinsichtlich der Verteilung von Geschlecht, Wohnsituation und Familienstand auf. Etwas mehr als die Hälfte der Probanden lebte allein und war entweder geschieden oder verwitwet. Die Ergebnisse der vorliegenden qualitativen Studie stehen zudem in Einklang mit den Ergebnissen einer großen repräsentativen Studie zur psychosozialen Gesundheit älterer Menschen in Deutschland [10].

## Fazit/Schlussfolgerung

Ältere Menschen erhalten sich überwiegend ihre psychosoziale Gesundheit während der COVID-19-Pandemie. Bei Personen mit bereits vor der Pandemie bestehenden psychosozialen Belastungen und Schwierigkeiten in der Alltagsbewältigung verschärfen sich diese gegebenenfalls im Zusammenhang mit einschränkenden Maßnahmen. Psychosoziale Hilfsangebote sollten daher insbesondere auf spezifische Subgruppen älterer Menschen ausgerichtet werden. Vor dem Hintergrund von Einsamkeit als überdauerndem Problem sind eine vollständige Schließung seniorenspezifischer Treffpunkte sowie Besuchsverbote in Pflegeheimen zu überdenken. Ältere Menschen wünschen sich eine zumindest teilweise Aufrechterhaltung solcher Kontaktorte unter Beachtung geeigneter Hygienemaßnahmen. Die Bedeutsamkeit mentaler Ressourcen älterer Menschen für die psychosoziale Unterstützung von Peers und jüngeren Generationen scheint bisher zu wenig Berücksichtigung zu finden. Lebenserfahrungen älterer Menschen sollten als gesellschaftliche Ressource verstanden und besser genutzt werden. Dies kann zum Beispiel durch eine stärkere Einbindung älterer Menschen in die Gestaltung und Umsetzung psychosozialer Unterstützungsangebote erfolgen.

## Supplementary Information


