# Supplementary material for: Health-related consequences of social isolation: a qualitative study on psychosocial stress and resources among older adults in the COVID-19 pandemic
Source: Bundesgesundheitsblatt Gesundheitsforschung Gesundheitsschutz. 2021 Feb 2;64(3):334–41. [Article in German] doi: 10.1007/s00103-021-03281-5 (PMC7852481; doi:10.1007/s00103-021-03281-5)
Supplement: Supplementary file 1 [file 103_2021_3281_MOESM1_ESM.pdf]

Bundesgesundheitsblatt 3/2021

Elektronisches Zusatzmaterial zum Beitrag:

Gesundheitliche Folgen sozialer Isolation: Qualitative Studie zu psychosozialen Belastungen und Ressourcen älterer Menschen im Zusammenhang mit der COVID-19-Pandemie

Franziska D. Welzel[1], Katja Schladitz[1], Franziska Förster[1], Margrit Löbner[1], Steffi G. Riedel-Heller[1]

[1]Institut für Sozialmedizin, Arbeitsmedizin und Public Health (ISAP), Medizinische Fakultät, Universität Leipzig, Leipzig, Deutschland

Korrespondenzadresse:
Franziska D. Welzel
Institut für Sozialmedizin, Arbeitsmedizin und Public Health (ISAP),
Universität Leipzig,
Philipp-Rosenthal-Straße 55,
04103 Leipzig
Franziska.Welzel@medizin.uni-leipzig.de

Inhalt:

Tabelle Z1. Übersicht über die Schlüsselzitate der Interviewteilnehmer/innen.

Tabelle Z1. Übersicht über die Schlüsselzitate der Interviewteilnehmer/innen.
HK = Hauptkategorie; UK = Unterkategorie.

**HK1: Infektions- und Isolationserfahrungen**	**UK1: Infektions-erfahrung**	„Wir fragen immer rum, ob jemand jemanden kennt, aber wir kennen noch nicht einmal jemanden, der jemanden kennt.“ (200513_0002.MP3 – Transkript – Cor04: 30)
	**UK2: Isolationserfahrung**	„Sonnabend entlassen, Dienstag kriegte ich dann Bescheid vom Gesundheitsamt, ich wäre in Quarantäne. Ach (lacht), naja, das ist natürlich/irgendeine Schwester, die mit mir im Gange war, von dort kommt das her. Naja, da kam dann auch/die haben öfters angerufen, waren sehr nett, vom Gesundheitsamt, dann kam jemand und guckte, was weiß ich, ob ich da bin oder so. Ich musste sogar den Ausweis zeigen.“ (200617_0041.MP3 – Transkript – Cor20: 4)
**HK2: Bewertung des COVID-19-Geschehens**	**UK1: Zustandsbewertung**	„Also ganz ehrlich gesagt, natürlich waren wir erst ein bissel erschrocken, und ja, und verunsichert und/und das zwar ganz schön (lachen). ABER ja, im Laufe der Zeit hat sich das dann schon bissel gebessert oder normalisiert.“ (200619_0010.MP3 – Transkript – Cor09: 4)
	**UK2: Ängste/Sorgen**	**Individuell:** „Ja, früher starb man schneller, das war angenehmer, weil man nicht die ganzen Krankheiten, die im Alter kommen, bis zum Ende ausleben muss und die ja auch oft von Medikamenten erst verursacht werden. Und nun müssen wir eben auch noch die Corona-Krankheit mehr oder weniger, ja, was willst du machen. Wenn wir sie haben, dann haben wir sie, wenn nicht, dann nicht. Ich schreie natürlich nicht ‚hier, ich will sie haben‘, aber da ist so ein Fatalismus drin, vielleicht, kann man auch nicht sagen.“ (200617_0041.MP3 – Transkript – Cor20: 12)
		**Familie & Bekannte:** „Und von meiner Familie hab ich das ja auch, die Probleme, die da auftreten, aber ich bin ja hier alleine und hab da nicht so viele Probleme, wie zum Beispiel meine Enkelin, die zwei kleine Kinder hat und bei L. im Schichtdienst arbeitet und der Mann im Steuerbüro und auch kein Homeoffice machen kann und sie ihre zwei kleinen Kinder nicht untergebracht hat und sie dann zu meiner Tochter nach D. schaffen musste, obwohl das ja eigentlich gar nicht erlaubt war, dass man jetzt hier Sachsen verlässt. Da mache ich mir dann schon mehr Gedanken, als was mich selber persönlich betrifft.“ (200515_0017.MP3 – Transkript – Cor17: 22)
		**Gesellschaftlich:** „Beschäftigen tut ich mich eben mehr nicht meine persönliche Einschränkung, die ich eventuell habe, die ich kaum vermisse, sondern die allgemeine Situation in der Wirtschaft, die ist für mich sehr maßgebend. Das hab ich am Anfang schon befürchtet, aber dass das so schlimm kommt, hatte ich nicht erwartet, weil ja das ganze Wirtschaftsleben im Prinzip fast zusammen bricht. Die Einschränkung, die ich in der persönlichen Freiheit hab, wie Sport oder Kultur, vermisse ich nicht, aber die Leute, die das organisieren müssen, ob das hier Sport- oder Kulturveranstaltungen sind, die leben ja an der der Existenzgrenze, und das ist natürlich extrem schwer.“ (200511_0004.MP3 – Transkript – Cor05: 4)
	**UK3: Bewertung einschränkender Maßnahmen**	**Alltagseinschränkungen: „**Wo ich dann immer bisschen nervig werde ist eben beim Einkaufen mit der Maske. Ich bin Brillenträger, die beschlägt ja ständig, wenn ich im Laden bin und so. Und da mache ich nur das Nötigste. Das, was ich kaufen wollte oder was ich brauche und dann so schnell wie möglich wieder raus. Also so geht es mir und da hab ich keine Lust mehr.“ (200520_0038.MP3 – Transkript – Cor07: 40)
		**Soziale Kontakte:** „Tja, ja, klar war man etwas/ naja, ich würde auch gern mal wieder in eine Gaststätte gehen. Freisitz mit Freunden zusammen, denn das gehörte eigentlich auch zu unserem Ablauf der ehemaligen Kollegen, dass wir uns eigentlich regelmäßig getroffen haben, um uns gegenseitig auch unsere

		Krankheiten zu beichten. Aber trotzdem war es immer schön (lachen) und ich vermisse das eigentlich auch.“ (200604_0008.MP3 – Transkript – Cor21: 89)
		**Freizeit- & Sportaktivitäten:** „Und da war ich 2019 ziemlich am Boden und hatte mich dann gerade so bisschen rausgerappelt und mir ein anderes Umfeld gesucht, wo ich mich beschäftigen kann. Da war ich bei der Volkssolidarität und habe Vorträge mit wahrgenommen und bin da dorthin gegangen und habe montags auch Rommé gespielt und zum Sport gegangen und zum Gedächtnistraining. Das ist ja nun auch alles weggebrochen.“ (200515_0017.MP3 – Transkript – Cor17: 16)
		**Urlaub & Reisen:** „Naja, es kommt ja jetzt die Urlaubszeit. Das mit dem Reisen, dass man dann nicht überall hinfahren kann und und und. Wir wissen es ja selber noch nicht. Wir wollten dieses Jahr Familienurlaub hier auf Mallorca machen. […] Aber ob wir im August dann fahren können, ist ja noch gar nicht raus, was in Spanien dann los ist. Und ob das dann/ mit Maske Urlaub ist ja dann auch nicht so schön. Oder mit Einschränkungen und und und. Da wissen wir ja auch noch nicht, was es wird. Das ärgert uns schon, weil alles ungewiss ist.“ (200520_0038.MP3 – Transkript – Cor07: 38)
		**Medizinische Versorgung:** „Nein, nein. Das kann ich natürlich leicht sagen, weil ich ja gesund bin. Und wenn wir irgendwelche Probleme hätten, würden wir beim Arzt vorher anrufen und fragen, ob wir kommen können.“ (200507_0003.MP3 – Transkript – Cor08: 48)
		**Finanzielle Einschränkungen:** „Finanziell nein, nein, überhaupt nicht. Im Gegenteil, ich muss Ihnen ganz ehrlich sagen, ich hab jetzt so viel Geld gespart (Lachen) wie noch nie von meiner Rente in den letzten 20 Jahren. Nein wann, seit wann bin ich in Rente, seit/ also seit ich in Rente bin. Das kann ich Ihnen auch sagen, warum. Ich gehe ja NICHT mehr in kulturelle Veranstaltungen, ich gebe kein Geld mehr aus für Veranstaltungen, für Kino, für Konzerte, für Theater. […] Also ich hab noch nie so viel gespart wie jetzt.“ (200511_0037.MP3 – Transkript – Cor16: 38)
**HK3: Bewertung vorhandener Ressourcen**	**UK1: Eigene Ressourcen**	**Lebenserfahrung:** „Ja, Lebenserfahrung. Na, ich denke jetzt auch viel an früher, dass/ Ich bin ja nun ´40 geboren, im Krieg aufgewachsen und als Kind hatte meine/ meine Schwester ist ´44 geboren und meine Mutti war dann alleine mit uns, weil mein Vati erst ´47 aus der Gefangenschaft gekommen ist und da hatten wir nichts zu essen. Das finde ich jetzt/ Jetzt geht´s uns ja gut, jetzt haben wir ja alles im Überfluss. Und wenn ich an die Zeit denke… das find ich jetzt, dass manche sich da beschweren, dass sie eingesperrt sind, aber nach dem Krieg, da ging es ja uns noch schlechter. Da waren wir zwar nicht eingesperrt, aber wir hatten ja gar nichts, erstens, dass wir unternehmen konnten oder das wir essen konnten. Da ging es uns ja viel schlechter. Und dann, als eben meine Kinder geboren wurden, die sind noch nicht mal ein Jahr auseinander, da hatte ich auch viel zu tun. Da hatten wir kein Telefon, wir hatten kein Auto. Ich musste viel zum Arzt mit den Kindern, und also das war eine fast noch eine schlimmere Zeit als jetzt, finde ich.“ (200515_0017.MP3 – Transkript – Cor17: 86)
		**Mentale Ressourcen**: „Also schön auf dem Teppich bleiben, Ruhe bewahren und das funktioniert eigentlich immer ausgezeichnet. Egal wie die Situation ist, es waren schon solche Situationen, wo man Todesangst haben musste, sowas – das hab ich nicht erlebt in meinem Leben. Toi toi toi. Aber Ruhe bewahren und dann, wie die Situation ist, ein bissel analysieren und dann versuchen, das Beste draus zu machen. Klappt's, haste Glück, klappt's nicht, haste Pech (Lachen).“ (200513_0016.MP3 – Transkript – Cor02: 78)
		**Wohnumfeld:** „Aber so konnte ich ja immer raus, konnte immer an die Luft gehen, und ob das hier durch den Wald oder dann hier im Garten war, wo man

		die Natur anguckt, das sind ja alles Sachen, die für das Wohlbefinden hier wichtig sind." (200511_0004.MP3 – Transkript – Cor05: 50)
	**UK2: Externe Ressourcen**	**Familiäre & freundschaftliche Hilfe:** „Naja, dass ich eben wirklich einen guten Bekanntenkreis auch habe. Also dass auch alles in der Familie klappt, eine intakte Familie habe, und einen super Bekanntenkreis habe. Wo man sich dann mal wirklich über Dinge, die einen so mal bewegen reden kann, und so, das hat mir SEHR geholfen. Aber das hilft mir auch in meiner Bewältigung des Alltags immer, SEHR. Na, weil das auch Sachen sind, also Freundschaften sind, die schon 40 Jahre halten." (200619_0010.MP3 – Transkript – Cor09: 59)
		**Nachbarschaftshilfe**: „Was ich gut finde, es war einmal gleich dann, wo das anfing, dann das ganze Theater, sag ich mal, das ist nicht negativ gemeint, da kam eine Studentin aus meinem Haus, hat bei uns geklingelt – mir gegenüber wohnt noch eine ältere Frau, wir sind die zwei ältesten hier – hat geklingelt und hat gefragt, ob sie uns was mitbringen kann und so. Das hat mich natürlich SEHR gefreut, denn man schimpft immer auf die Jugend, also da muss ich sagen, das hat mich sehr gefreut." (200513_0016.MP3 – Transkript – Cor02: 22)
		**Gesellschaftliche Hilfe & Dienstleistungen:** „Hab ich NICHT gemacht, aber DAS hätte man auch machen können, und das finde ich auch ganz toll. Das find ich ja auch so lieb und toll, wer da niemanden hat, dass man die schweren Flaschen und so wenigstens nicht schleppen muss, also diese Dienste da mit dem Durstdienst, und so, das find ich sowieso ganz großartig. Ne, auch ohne Corona. Na, und die Lieferdienste, das find ich auch super, also das ist wirklich eine tolle Sache." (200619_0010.MP3 – Transkript – Cor09: 55)
**HK4: Eingesetzte Bewältigungsstrategien**	**UK1: Aufrechterhaltung körperliches Wohlbefinden**	„Und naja gut, das Sportliche fehlt einem, was einem fehlt als soziale Kontakte hier, Fitnessstudio oder allgemeine Sportgruppe, wo ich immer hingegangen bin. Muss ich eben dann allein faktisch hier mal so eine Wanderung machen." (200511_0004.MP3 – Transkript – Cor05: 28)
	**UK2: Aufrechterhaltung seelisches Wohlbefinden**	„Um einen schönen Alltag zu machen. Also ich höre GANZ, GANZ viel Radio, MDR Kultur immer, also jeden Früh und die Lesezeit und das liebe ich geradezu. […] Also ich höre sehr viel, ich lese sehr viel und ja, und damit komme ich ganz gut zurecht." (200511_0037.MP3 – Transkript – Cor16: 44)
	**UK3: Rolle der Medien**	**Informationsquellen (klassisch & neu):** „Na, ich mache das im Prinzip nur über Radio, Zeitung und dann Fernsehen. Also, Internet weigere ich mich, da reinzugucken hier. Meine Frau, die guckt da ganz intensiv hier die ganzen Berichte, die da drinstehen und so hier, die machen einen ja nur verrückt. Da kommt man nur durcheinander, weil jedes einzelne Argument so eine gewisse Richtigkeit haben könnte, wo man dann stark ins Zweifeln kommt und so." (200511_0004.MP3 – Transkript – Cor05: 56)
		**Zwecke Internetnutzung:** „Zur Information. Zum Schreiben. Und Geldüberweisung, falls ich Geld habe (Lachen). Dann/ das mach ich eigentlich/ das tu ich mir nicht mehr an heutzutage dahinzugehen. Ich hab zwar eine um die Ecke hier, aber das kann man schön machen. Du kannst jeden Tag auf dein Konto gucken. Ja, das mach ich gerne." (200513_0016.MP3 – Transkript – Cor02: 96)
		**Aufrechterhaltung sozialer Kontakte:** „Na, WhatsApp sowieso, ja. WhatsApp mit allen Freunden, Verwandten, Bekannten und so. WhatsApp ist klar. Das ist ein wichtiges Medium geworden, muss ich ganz ehrlich sagen." (200619_0010.MP3 – Transkript – Cor09: 77)
		**Aufrechterhaltung medizinischer Versorgung:** „Ja, ich würde das mitmachen. Also ich würde dann eben diese Sprechstunden nutzen. Ich hab ja zum Beispiel ein Problem, diese Bluttabletten, also das sind Blutverdünner. Also ich schlafe ja nur noch, ich muss mich hinlegen, ist furchtbar. Die habe ich jetzt reduziert auf die Hälfte und da würde ich eben gern den Arzt fragen, ob er da einverstanden ist oder ob das hochgefährlich ist, weil es gibt auch Sachen, die müssen in einer bestimmten Menge zueinander passen. Ja. So, das kann ich alles nicht. Ich muss

		also bis zum nächsten Quartal warten, auch wenn ich dann zwei-, dreimal komme, da ist er auch böse, ist ja so, wissen wir ja auch alle. Und dann stelle ich mir so die leichten, einfachen Infokontakte gut vor.“ (200617_0041.MP3 – Transkript – Cor20: 96)
**HK5: Kohärenzsinn**	**UK1: Verstehbarkeit**	„Naja, im Moment, ja. Am Anfang war das natürlich ein bisschen Holterdiepolter, wo sie dann die Kriterien geändert haben. Aber das musste sich ja erst einspielen, das ist ja dann/ wie gesagt, am Anfang hieß es ja dann die Verdoppelungszeit zu zählen, oder dann die Reproduktionszahl. Und jetzt haben sie wohl eine neue Kennziffer rausgegeben mit so und so vielen Infizierten pro 100.000 Einwohnern pro Tag, oder pro Woche, das ist überzeugend jetzt, ja.“ (200507_0003.MP3 – Transkript – Cor08: 120)<br><br>„Ja, es wurde ja viel gesprochen, gerade in den Talkrunden da mit dem Virologen und und und. Ja es ist ja alles auch noch ein Suchen letztendlich und dann lieber etwas strenger sag ich, als eben zu locker und dann schnellt die Kurve dann wieder hoch und das will ja keiner. Das sehen sie ja in New York und hier in Spanien, Italien, wie das war und und und. So und da haben wir doch/ ist das lieber jetzt so, wie sie es gemacht haben richtig, finde ich richtig. Ja klar, da haben dann viele gemeckert und und und, aber ich finde das ist schon ok. Und es haben ja alle gelernt, es wusste ja keiner, so was gab es auch noch nie. Also ich wüsste es nicht in meiner ganzen Lebenszeit, dass schon so was Ähnliches mal war.“ (200520_0038.MP3 – Transkript – Cor07: 131)
	**UK2: Handhabbarkeit**	„Also, das mache ich und ich finde die Regeln in Ordnung und, obwohl die mir manchmal auch ein bisschen nicht passen, aber ich finde das in Ordnung, dass das so ist. Punkt. Und ich finde auch in Ordnung, dass es diese ganzen Maßnahmen gibt. Ja, das finde ich schon gut und ich propagiere das auch, ich propagiere das auch ringsum. Und sage, ‚Mensch Leute, kommt jetzt, nun setzt eure Maske auf und alles ist gut‘ und ja, ja, aber das ist auch das einzige, was ich dafür tun könnte, kann.“ (200511_0037.MP3 – Transkript – Cor16: 110)
	**UK3: Chancen der Krise/Bedeutsamkeit**	**Individuell:** „Mir würde es nichts ausmachen, wenn es auch weiterhin dabei bleibt, dass sie mir helfen, mich unterstützen, wenn ich Hilfe brauche.“ (200515_0017.MP3 – Transkript – Cor17: 168)
		**Familie & Bekannte:** „Naja, meine eine Nachbarin schon. Nicht immer, aber die ist von/ von ihrem Wesen sehr pessimistisch und/ und die zur Zeit öfter mal eben so ein Tief oder ein kleines Loch, und ganz ehrlich gesagt, die sagt immer ‚du fehlst mir‘, die rück ich mal gerade. […] JA, dann sag ich mein Gott, jetzt reiß dich mal zusammen, du lebst und sei froh, dass du auf dein Fahrrad steigen kannst und da dir die Leute angucken kannst. Also genieße das, und/ als Beispiel unter anderem (lachen). So dann muss ich die immer mal, und dann machen wir zusammen, und dann sagt sie ‚ach, das hab ich mal wieder gebraucht‘.“ (200619_0010.MP3 – Transkript – Cor09: 117–119)
		**Gesellschaftlich:** „Naja, eine Chance sehe ich, es gibt ja/ ist nur die Frage in welche Richtung das alles ausgeht. Ich meine, es sind ja so viele Verwerfungen in einer Gesellschaft, ob das/ ich rede jetzt mal speziell vom Fußball, wo ich mehr Einblick habe, als faktisch hier bei Kulturveranstaltungen so. Und da, die Gehälter in der Bundesliga, dass sich da eventuell was ändert. Aber ansonsten glaube ich nicht, dass die Gesellschaft sich groß verändert, weil, naja, das ist eben so. Immer größer, weiter, höher und so weiter. Die Moral sinkt immer mehr, und ob die besser wird, bezweifle ich. Also ich sehe eigentlich nicht, dass sich grundlegend etwas verändern wird. Nach einer gewissen Zeit wird das vergessen, sollte das wirklich hier irgendwann in den Griff kommen.“ (200511_0004.MP3 – Transkript – Cor05: 86)

**HK6: Versorgungs- & Unterstützungsbedarfe**	**UK1: Informationsbedarfe**	„Nee, ich finde die Medien, ich sage mal, Fernsehen und Radio berichten ja eigentlich stündlich. Und wer sich damit auseinandersetzen will, der hat die Möglichkeit, sich das anzuhören. Was man für eine Meinung sich dazu bildet, ist die eine Sache. Aber ich könnte nicht sagen, dass mir irgendwas verheimlicht worden wäre.“ (200604_0008.MP3 – Transkript – Cor21: 121)
	**UK2: Unterstützungsbedarfe**	„Ich kann mir nicht vorstellen wie es hätte sollen sein, die Unterstützung. Muss ich ganz ehrlich sagen. SICHERLICH, aber ich meine, die Einsamkeit und so für ältere Menschen, ich glaube, die ist auch ohne Corona sehr verbreitet. Bei uns hier in/ überhaupt in Deutschland. ABER ich denke mir mal, dass das immer ein Thema sein wird, auch ohne Corona. […] weil eben auch mehr darüber geredet wurde. Aber ich glaube, die hier in Corona-Zeiten richtig einsam waren, die waren's auch vorher schon.“ (200619_0010.MP3 – Transkript – Cor09: 105-107)<br><br>„Hab ich mir noch keine Gedanken gemacht, aber ich meine, das ist natürlich auch davon abhängig, in welchem persönlichen gesundheitlichen Zustand die älteren Leute sind. Wie gesagt, das Drittel der Leute, die in diesem Haus hier so im Grunde genommen sich nur abschotten – ich weiß nicht. Könnte ich mir nicht vorstellen, dass man da unbedingt die Hilfe erhöhen könnte, weil die wollen es eigentlich gar nicht. Und wenn welche sind, die Hilfe brauchen – ich denk mal, da gibt es in der Nachbarschaft trotzdem Leute, die das tun, ohne viel Aufsehen.“ (200513_0002.MP3 – Transkript – Cor04: 146)
